# Metabolic Dysfunction-Associated Fatty Liver Disease (MAFLD)—A Condition Associated with Heightened Sympathetic Activation

**DOI:** 10.3390/ijms22084241

**Published:** 2021-04-19

**Authors:** Revathy Carnagarin, Kearney Tan, Leon Adams, Vance B. Matthews, Marcio G. Kiuchi, Leslie Marisol Lugo Gavidia, Gavin W. Lambert, Elisabeth A. Lambert, Lakshini Y. Herat, Markus P. Schlaich

**Affiliations:** 1 Dobney Hypertension Centre, School of Medicine—Royal Perth Hospital Unit, RPH Research Foundation, Faculty of Medicine, Dentistry and Health Sciences, The University of Western Australia, Perth, WA 6000, Australia; revathy.carnagarin@uwa.edu.au (R.C.); krnytjy@gmail.com (K.T.); vance.matthews@uwa.edu.au (V.B.M.); marcio.galindokiuchi@uwa.edu.au (M.G.K.); lesliemarisol.lugogavidia@research.uwa.edu.au (L.M.L.G.); lakshini.weerasekera@uwa.edu.au (L.Y.H.); 2 Medical School, Faculty of Medicine, Dentistry and Health Sciences, The University of Western Australia, Perth, WA 6009, Australia; leon.adams@uwa.edu.au; 3 Iverson Health Innovation Research Institute and School of Health Sciences, Swinburne University of Technology, Melbourne, VIC 3122, Australia; glambert@swin.edu.au (G.W.L.); elisabethlambert@swin.edu.au (E.A.L.); 4 Human Neurotransmitter Lab, Melbourne, VIC 3004, Australia; 5 Neurovascular Hypertension and Kidney Disease Laboratory, Baker Heart and Diabetes Institute, Melbourne, VIC 3004, Australia; 6 Departments of Cardiology and Nephrology, Royal Perth Hospital, Perth, WA 6000, Australia

**Keywords:** sympathetic nervous system, metabolic syndrome, hepatic denervation, multi organ denervation

## Abstract

Metabolic dysfunction-associated fatty liver disease (MAFLD) is the most common liver disease affecting a quarter of the global population and is often associated with adverse health outcomes. The increasing prevalence of MAFLD occurs in parallel to that of metabolic syndrome (MetS), which in fact plays a major role in driving the perturbations of cardiometabolic homeostasis. However, the mechanisms underpinning the pathogenesis of MAFLD are incompletely understood. Compelling evidence from animal and human studies suggest that heightened activation of the sympathetic nervous system is a key contributor to the development of MAFLD. Indeed, common treatment strategies for metabolic diseases such as diet and exercise to induce weight loss have been shown to exert their beneficial effects at least in part through the associated sympathetic inhibition. Furthermore, pharmacological and device-based approaches to reduce sympathetic activation have been demonstrated to improve the metabolic alterations frequently present in patients with obesity, MetSand diabetes. Currently available evidence, while still limited, suggests that sympathetic activation is of specific relevance in the pathogenesis of MAFLD and consequentially may offer an attractive therapeutic target to attenuate the adverse outcomes associated with MAFLD.

## 1. Introduction

**Figure 1:**
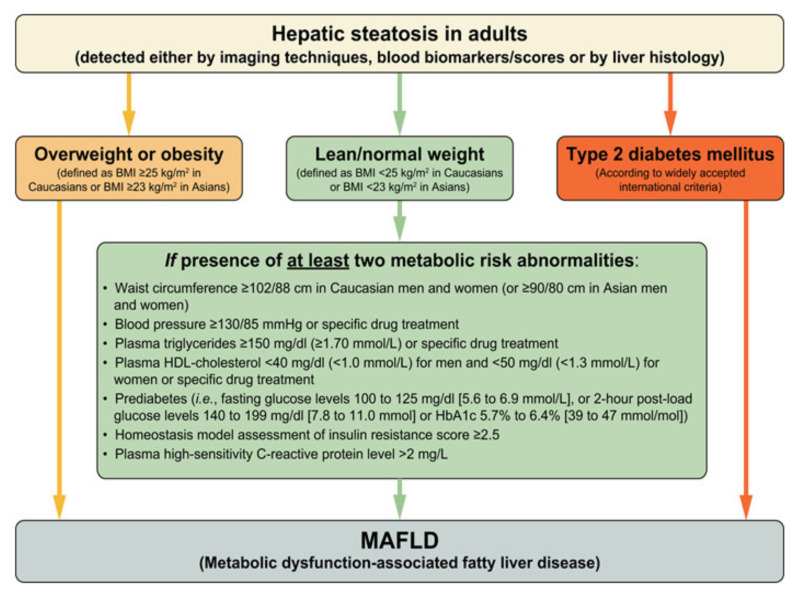
Flowchart for the proposed “positive” diagnostic criteria for MAFLD (Reprinted from [7] with permission from Elsevier’).

Metabolic dysfunction-associated fatty liver disease (MAFLD) has become the most common chronic liver condition in developed and developing countries due to the burgeoning in the incidence of obesity and metabolic syndrome(MetS) [1]. MAFLD was previously identified as non-alcoholic fatty liver disease defined as the accumulation of excess fat in hepatocytes, in the absence of secondary causes of steatosis such as excess alcohol intake (30 g/day for men and 20 g/day for women) [2,3]. The MAFLD spectrum ranges from simple steatosis to steatohepatitis and ultimately development of fibrosis and cirrhosis in the long term with an increased risk of hepatocellular carcinoma [4,5]. Importantly, as many as 30% of subjects with fatty liver disease have histologic evidence of liver inflammation, which in turn is associated with increased risk of progression to cirrhosis [6]. Recently, an international expert consensus statement coined the new definition of fatty liver disease associated with metabolic dysfunction, MAFLD [7], to extend the perspective of disease assessment and severity stratification beyond a simple dichotomous classification of steatohepatitis vs. non-steatohepatitis. Moreover, this was based on the evidence of hepatic steatosis, in addition to one of the following three criteria: overweight/obesity, diabetes mellitus or evidence of metabolic dysregulation, as summarized in Figure 1 [7].

**Figure 2:**
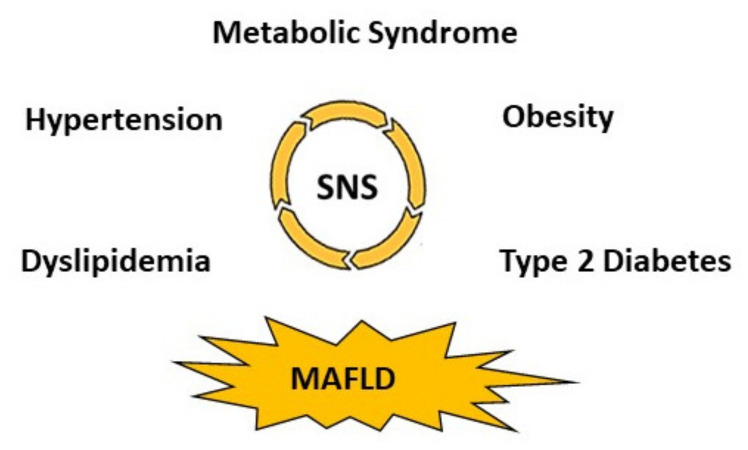
Sympathetic overdrive is the central pathogenesis of metabolic disorders.

The pathophysiological mechanisms underlying the MetS are complex and extend beyond sedentary lifestyle, poor diet and genetic predisposition. It is now becoming clear from some of our own observations and those of others that the sympathetic nervous system (SNS) is important in the generation of both obesity and obesity-related illness [8]. Of growing interest is the interaction between the SNS and the liver in the development and clinical consequences of the MetS. Indeed, the link among glucose tolerance, insulin sensitivity and liver function is evident, with MAFLD being the hepatic manifestation of the MetS. The pathogenesis of MAFLD is incompletely understood and currently no proven therapy exists that addresses both the progression of liver fibrosis and the associated metabolic disturbances. There is strong evidence implicating SNS activation in the pathogenesis of cardio-metabolic illnesses including obesity, MetS, diabetes, hypertension (HTN) and other conditions including MAFLD (Figure 2). A complex interplay of endocrine mechanisms, immune activation, microbial dysbiosis, etc. can perpetuate SNS hyperactivity across the metabolic disease continuum [9,10,11,12], resulting in unwanted consequences such as insulin resistance and systemic inflammation [13,14,15].

Investigators have reported clear evidence that liver fibrogenesis and MAFLD are associated with heightened sympathetic activation [16]. Sympathoexcitation—as indicated by an increase in circulating neurotransmitters such as norepinephrine (NE) and neuropeptide Y (NPY)—has been identified to induce hepatic stress and fibrogenesis in hepatic stellate cells (HSC) at physiological concentrations, in both animal models and humans [16,17,18]. Furthermore, inhibition of the SNS increased hepatic progenitors and reduced liver injury [19]. Additionally, noradrenergic antagonism inhibited fibrogenesis in rat livers [19,20]. Moreover, weight loss mediated attenuation of sympathetic overdrive promoted improvement in metabolic parameters and liver enzymes in obese-hypertensive patients [21,22]. Sympatholytic agents such as α2-receptor agonists and I1 Imidazoline receptor agonists, rilmenidine or moxonidine demonstrated similar effects [23,24,25]. However, whether these effects are definite in improving aspects of MAFLD remains to be seen. This article reviews the current literature from PubMed on the association of sympathetic activation with MAFLD and discusses the potential treatments targeting the sympathetic overdrive that may benefit the patients with MAFLD.

## 2. Metabolic Dysfunction Associated Fatty Liver Disease (MAFLD)

The onset of MAFLD begins with benign steatosis with the accumulation of triglycerides (TG) in the hepatocytes, which, if not reversed, progresses to non-alcoholic steatohepatitis [26]. Previous studies have shown that, in patients with MAFLD, 60% of hepatic TG accumulation is derived from circulating free fatty acids (FFA), 25% from de novo lipogenesis and 15% from diet [27]. Steatohepatitis is characterized by lobular inflammation, hepatocyte ballooning, fibrosis and cirrhosis [26,28]. Approximately one third of patients with MAFLD progress to steatohepatitis and a smaller percentage of that group progresses to cirrhosis, which may transform to hepatocellular carcinoma [29].

With only a proportion of patients progressing towards steatohepatitis, fibrosis and cirrhosis, the factors responsible for the development of MAFLD are yet to be determined [5]. The traditional theory of MAFLD progression is the “two-hit theory” put forward by Day and colleagues [30]. The first step is the accumulation of hepatic triglycerides, which occurs when the rate of lipid disposal is exceeded by the influx of the free fatty acids (FFA) [31]. Additional mechanisms such as insulin resistance (IR), increased dietary influx and increased hepatic lipogenesis (via de novo lipogenesis) on top of genetic predisposition to metabolic abnormalities enhance hepatic FFA trafficking [32]. Furthermore, in insulin resistant states, the inability of insulin to suppress adipolysis results in increased hepatic triglyceride synthesis by de novo lipogenesis along with hyperinsulinemia [33]. This results in elevated FFA, free cholesterol, oxidized cholesterol metabolites and other toxic metabolites that can act as reactive oxygen species [26,34], thereby creating a lipotoxic atmosphere for the hepatocytes. The second step is characterized by lipotoxic oxidative stress in the hepatocytes [26] resulting in hepatocellular mitochondrial dysfunction and endoplasmic reticulum (ER) stress that further potentiate the oxidative stress cascade [26,34]. This cascade of events results in chronic hepatocellular inflammation, apoptosis and eventually hepatic fibrosis [34] that eventually progress to complications such as hepatocellular carcinoma (HCC) and portal hypertension [35].

However, the “two-hit” hypothesis is now obsolete due its inability to explain the multitude of molecular and metabolic changes that take place in MAFLD. Hence, the “multiple hit” hypothesis was proposed, which states that, when genetically predisposed subjects are exposed to multiple epigenetic insults, their liver injury may proceed to develop MAFLD. This hypothesis emphasizes that the pathogenesis and progression of MAFLD is very complex and involves coherent interplay of multiple factors such as insulin resistance, abnormal hormonal secretion, obesity, diet, genetic factors, immune activation and, as shown more recently, gut dysbiosis that work in parallel to cause the progression of MAFLD [34,36,37].

## 3. Sympathetic Nervous System (SNS) Activation, Metabolic Dysregulation and MAFLD

**Figure 3:**
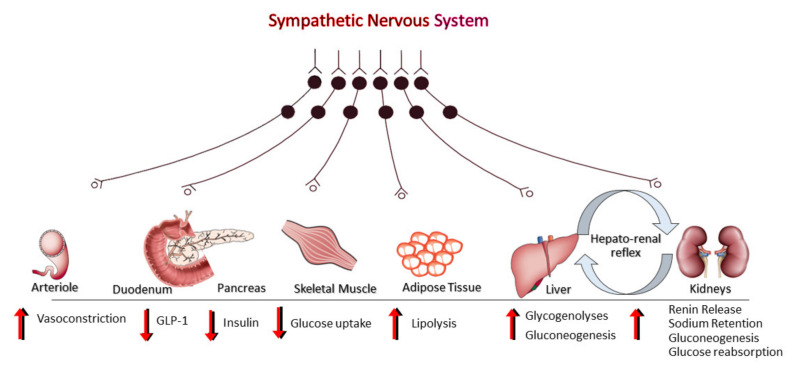
Overview of the effect of sympathetic activation on metabolic pathways and the sustenance of sympathetic tone through the hepato-renal reflex and the adverse impacts on cardiometabolic regulation.

The sympathetically mediated metabolic effects (Figure 3) enable the human organism to cope with stressful situations of short duration when there is a need for increased energy requirements. However, sustained sympathetic overdrive often results in adverse cardiovascular and metabolic consequences, potentially including MAFLD. Sympathetic stimulation of the hepatic nerves [38] induces a rapid and marked glucose output from the liver, whereas stimulation of pancreatic sympathetic nerves is associated with reduced insulin and increased glucagon secretion into the portal circulation [9]. Furthermore, enhanced sympathetic activation results in neurally mediated peripheral vasoconstriction in skeletal muscle [8], associated with impaired glucose uptake and insulin resistance and enhanced lipolysis in adipocytes. This lipolytic state induced by the sympathetic overactivity results in increased levels of free fatty acids and triglycerides in the circulation and visceral deposition, exacerbating MAFLD in patients with metabolic diseases [39,40,41].

MetS is a sympathetic disease [9] and has been found to be a strong predictor of MAFLD [42]. The endocrine and biochemical disturbances that characterizes MAFLD are associated with increased sympathetic activity [41,42]. SNS mediated insulin resistance, influenced by hypothalamic neuropeptide Y and other factors in turn results in compensatory hyperinsulinemia and hyperglycemia in metabolic disease states, thereby resulting in the progression of MAFLD [43,44,45]. Heightened sympathetic activation has been demonstrated using gold standard techniques such as microneurography and nor-adrenaline spillover from whole body and from individual organs in patients with liver cirrhosis [46] and metabolic diseases [13,47]. Patients with perturbed glucose metabolism and obesity have accompanying liver cirrhosis, the progression of which results in progressive sympathetic activation mediated by disturbed cardiovascular and metabolic homeostasis and vice versa [46,47] In addition, various adipokines such as leptin and adiponectin are also known to affect the progression of MAFLD. Leptin has been shown to cause fibrogenesis in animal models [48,49] and it is known to prevent lipid accumulation, perhaps contributing to the compensatory hyperleptinemic status seen in obese patients [50]. Adiponectin has anti-inflammatory effects, improves insulin resistance and prevents hepatic damage by blocking the IKK-NF-κB inflammatory pathways, often downregulated in obesity [50]. Furthermore, enhanced fibrogenesis has been demonstrated in adiponectin deficient animal models [51]. Combined hypoadiponectinemia and hyperleptinemia in obesity facilitate the exacerbated progression of MAFLD [52]. Besides, hyperleptinemia is sympathoexcitatory due to its ability to cross the blood–brain barrier and modulate the sympathetic brain centers via the leptin receptors and is causally linked to hypertension in obesity and metabolic syndrome [53].

Moreover, nutrient excess in MetS and obesity warrants exaggerated mesenteric oxygen demand, reducing portal vein oxygen and hepatic oxygen delivery. The hepatic arterial buffer response, mediated by an adenosine induced arterial vasodilatory response, sustains a stable hepatic vein outflow despite the increase in post-prandial portal inflow. Eventually, adenosine is washed out by the increased postprandial portal flow restoring arterial resistance [54]. Chronic portal oxygen depletion limits adenosine triphosphate (ATP) production and induces adenosine release that further increases the hepatic sympathetic tone, triggering the “hepato-renal reflex”. Increased sympathetic activation in the liver results in the reduction of blood flow and increase in hepatic arteriolar resistance, which causes reflex renal sympathetic activation, with consequent renal arteriolar vasoconstriction and renin-angiotensin-aldosterone system (RAAS) activation [55]. The hepato-renal reflex mediated RAAS activation further decreases the renal blood flow, glomerular filtration and increases systemic sodium retention. The increase in afferent hepatic sympathetic activity is mediated by the low-pressure hepatic baroreceptors and which upon activation subsequently increases the cardiopulmonary and renal efferent sympathetic discharge, albeit without a change in heart rate [56].

The peri-venous hepatocellular lipid accumulation in MAFLD, compromises the oxygen delivery to the hepatocytes [55]. In addition, the low ATP status in MetS also contributes to metabolic inflexibility and impaired β-oxidation and further accentuates the hepatic lipid accumulation in MAFLD. Likewise, the onset of steatohepatitis in MAFLD further impedes the hepatic microcirculation sustaining hepatic hypoxia, inducing ATP/AMP depohosphorylation mechanisms that trigger the hepato-renal reflex [54,57]. A similar observation was made in high fructose induced MetS animals, where ATP depletion enriched adenosine production. Hepatocellular fructose metabolism is exclusively mediated by fructokinase that consumes the inorganic phosphate from the ATP and forms fructose-1-phosphate, adenine nucleotide and uric acid, which is a very sensitive indicator of hepatic ATP depletion [58]. In a clinical scenario, diabetic patients do not tolerate intravenous fructose challenge in large doses due to impaired ATP recovery [59]. Additionally, the metabolic surgeries performed in morbid obesity either bypasses or minimize nutrient contact, lowering splanchnic oxygen demand and enhances portal oxygen availability. This resulted in increased ATP and reduced uric acid production which blunts the hepato-renal reflex and the sympathetic activation following bariatric surgery [55,60].

## 4. Sympathetic Activation and MAFLD Progression

The exact mechanism that links sympathetic activation with the development of MAFLD remains to be determined. Besides the external factors linked with sympathetic activation, it has also been shown that SNS activity may directly influence the hepatic stellate cells (HSCs) in the pathogenesis of MAFLD [59]. Liver fibrosis results from repeating cycles of hepatocellular damage and repair, which transdifferentiates quiescent HSCs into a myrofibroblast phenotype characterized by increased secretion of the extracellular matrix protein such as collagen [61,62]. HSCs are the main source of collagen production and are located in close proximity with the sympathetic nerve fibers in the human liver [18,19]. HSCs are hepatic neuroglia that have been shown to express SNS receptors, namely α1A, α2B, β1, β2 and β3 adrenergic and NPY receptors [18,19], and the key enzymes for norepinephrine (NE) synthesis and release, namely dopamine-β-hydroxylase and tyrosine hydroxylase, all of which were upregulated in cirrhotic NAFLD human livers [16,19]. Additionally, the α- and β-adrenoceptor antagonists exerted an inhibitory effect on the growth of HSCs in various animal experiments [19]. These results are suggestive of sympathetic modulation of the HSCs through autocrine and paracrine mechanisms. Similarly, the NE deficient mice models demonstrated reduced proliferation of HSCs, compared to the controls and exogenous NE administration induced a dose-dependent (1 nM to 1 mM), biphasic, exacerbation of HSC proliferation [16]. In the same study, both exogenous and endogenous NE exerted an anti-apoptotic and proliferative effect on the human HSCs, whereas the other neurotransmitters such as the epinephrine and NPY exerted only proliferative effects on human HSCs [16]. Besides proliferation, adrenergic agonists (NE and isoprenaline) induced hepatic expression of transforming growth factor beta-1 (TGF-β1) and collagen genes in murine and human HSCs indicative of stellate cell activation [16].

## 5. Insights from Experimental Studies: Hepatic Fibrosis Is Sympathetically Driven

Further insights into the role of SNS in hepatic fibrosis was achieved by studying the role of NE in leptin deficient ob/ob mice, which display reduced NE levels, decreased SNS activity and was resistant to hepatic fibrosis [63,64,65]. The ob/ob mouse model has been shown to have reduced HSC levels and NE treatment induced HSC proliferation, upregulation of hepatic TGF-β1 and collagen augmenting liver fibrosis. [63]. Leptin was identified to play a key role in hepatic fibrogenesis due to its mitogenic impact on the HSCs via sympathoexcitation mediated by endogenous sympathetic neurotransmitters [66,67], affirming the notion that sympathetic activation drives leptin mediated hepatic fibrogenesis [66,67,68,69]. Moreover, dopamine β-hydroxylase deficient (Dbh−/−) mice displayed inhibited fibrogenic response to liver injury, as evidenced by decreased proliferation and activation of the HSCs [18]. In humans HSCs, NE stimulated the key intracellular pro-inflammatory pathways NF-κB, JNK/AP-1 and ERK, enhancing the secretion of inflammatory cytokines and chemokines such as IL-8 and RANTES, in a NF-κB dependent fashion [70,71]. Furthermore, this study also showed that NE stimulated the calcium release in human HSCs suggesting that NE could potentially influence HSC contractility, thus connecting the SNS to portal hypertension in cirrhotic livers [70].

Conversely, in vitro studies have proposed a regenerative role of catecholamines in the heterologous regulation of epidermal growth factor (EGF) receptors to aid hepatic regeneration [71]. Additionally, splanchnicectomy stimulated DNA synthesis and proliferation of hepatocytes following partial hepatectomy [72]. The facilitation of liver regeneration following partial hepatectomy was identified as parasympathetic vagal response, achieved by sympathetic inhibition through ventromedial hypothalamic lesions (sympathetic region) [73]. Moreover, hepatic branch sympathectomy [74] and bilateral subdiaphragmatic splanchnicectomy [72] produced no effect on liver regeneration.

## 6. Potential Therapeutic Implications

### 6.1. The Sympathetic Nervous System as a Target for Therapy

At present, the main treatment goal in patients with MAFLD is to manage individual risk factors such as obesity, high blood pressure and impairment in glucose and lipid metabolism. We summarize the available options for MAFLD and then explore the potential of direct sympathetic modulation as a therapeutic strategy.

### 6.2. Weight Loss and Exercise

With the increasing prevalence of MAFLD and related metabolic diseases, development of effective treatments is of utmost priority. Current approaches include lifestyle modification; specifically, weight loss via consistent exercise regimens or dietary restrictions and cognitive behavior therapy have been recommended as the preferred form of treatment for MAFLD [75,76]. Numerous studies exploring lifestyle interventions involving either exercise or calorie restriction or both that has resulted in a net weight loss, reduced abdominal and liver fat and improved insulin sensitivity have been associated with improvement of MAFLD [77,78,79,80,81]. Conversely, lower physical fitness has been associated with increased severity of MAFLD [82]. Both aerobic and resistance training have been shown to be effective but may depend on patients’ preferences to ensure commitment to the exercise regime to render it effective [79]. Multidisciplinary approaches addressing “psychosocial needs and behavioral support” may be effective [83] but were often influenced by personality factors and mental health issues such as depression, low consciousness and neuroticism in patients with MAFLD [84]. The reality is that adhering to lifestyle interventions is a strategy more easily advocated than practiced and patients do struggle with committing to such a drastic changes in lifestyles [85].

For severely obese patients, bariatric surgery might be an alternative option to achieve significant weight loss, which has also been associated with significant improvement and resolution of hepatic steatosis and fibrosis in a majority of the patients with body weight changes [86,87]. The evaluation of the beneficial hepatic changes in fibrosis with bariatric surgery are based on observational studies and the effect varies according to the type of surgery performed with a greater effect with bypass procedures compared to gastric banding [86]. Moreover, some cases were associated with progressive fibrosis and rarely fulminant steatohepatitis in the first postoperative year, presumably due to exaggerated weight loss following bypass surgery [86,87]. In MAFLD patients with cirrhosis, bariatric surgery may be contraindicated, in otherwise eligible morbidly obese subjects [88]. Therefore, pharmacological therapies may therefore represent the most accessible type of therapy for those that fail adherence to a strict regimen of exercise and, or dietary requirements [82].

## 7. Pharmacotherapy

Although there is an increasing demand for pharmacological therapies for patients with MAFLD, there is no specific form of treatment currently, which can meet adequate safety and efficacy standards [77]. Most treatments that have been studied target the various metabolic abnormalities that are associated with MAFLD [75]. Some examples include the Peroxisome Proliferator-Activated Receptors (PPAR) agonists known as thiazolidinediones, insulin sensitizing agents such as metformin, antioxidants such as vitamin E, a lipophilic antioxidant and the use of omega-3 polyunsaturated fatty acids, lipid lowering medications such as fibrates and statins [75,76], as summarized in Table 1.

In addition to weight reduction and exercise, pharmacological inhibition of the sympathetic nervous system might be a rational therapeutic approach for MAFLD and the associated metabolic perturbations [9]. Targeting sympathetic overdrive via weight loss has been shown to improve cardiometabolic abnormalities and markers of liver damage in hypertensive patients [21,22]. Lowering sympathetic activity with α-adrenergic blockers improved glucose and lipid profile along with blood pressure reduction [108,109]. However, the use of β-blocker drugs in obese people may be problematic. Investigations done from 1986 to 1998 clearly demonstrated the association of β blockers such as metoprolol, atenolol and propranolol with weight gain, worsening of insulin resistance and lipid profile, thereby enabling the progression of Mets patients to develop diabetes [110]. By contrast, the GEMINI trial showed that the use of carvedilol did not lead to substantial weight gain [111] and in fact was associated with beneficial effects such as improved insulin resistance [112], lipid profile [113] and a large reduction in microalbuminuria [114]. Moreover, the highly cardioselective, third-generation β blocker nevibolol demonstrated significant reduction in inflammation, improvement in lipid profile and blood glucose compared to conventional beta blockers [115].

Imidazoline I1 receptor agonists are antihypertensives that act centrally, at the level of the rostral ventrolateral medulla, to inhibit sympathetic activation. Besides lowering blood pressure, which is comparable to the antihypertensives of other classes, drugs such as moxonidine has demonstrated improvement in insulin sensitivity, glucose metabolism and dyslipidemia [116,117] and were associated with regression of target organ damage as evidenced by improved endothelial function [118], reduced left ventricular hypertrophy [119], renal function and reduction in microalbuminuria [120]. The combination of moxonidine with a weight loss program was shown to exert beneficial effects on aspects of the metabolic profile and end organ damage in young overweight males [121]. In a real-world primary care setting of patients with overweight/obesity-related BP elevation and metabolic disturbances (MERSY study), adding moxonidine to the treatment regimen resulted not only in the reduction of BP but also improved weight and metabolic profile [120]. Additionally, in a small, randomized, open parallel study in obese hypertensive subjects comparing the effects of moxonidine (0.2–0.4 mg/day, *n* = 19) and amlodipine (5–10 mg/day, *n* = 21) as a stand-alone therapy, moxonidine significantly reduced supine and orthostatic arterial and venous plasma noradrenaline levels and as well as reduced the leptin and insulin levels following 120 min after a glucose load [122]. Another highly relevant link in the current context is the sodium glucose co-tranporters-2 (SGLT-2) mediated sympathoinhibition and reduction in circulating noradrenaline, which has not only been shown to improve glucose control but, perhaps more importantly, also to reduce CV events in patients with type 2 diabetes [123,124]. Nevertheless, whether these drugs should be used preferentially over other drug classes in patients with MAFLD, MetS and obesity-related hypertension remains to be shown.

## 8. Device-Based Approaches

In addition to from lifestyle interventions and pharmacotherapy, device-based approaches are used to achieve sympathetic inhibition not only in the management of hypertension and its cardiovascular complications but also in metabolic conditions associated with sympathetic overdrive such as type 2 diabetes and MetS [125]. Catheter-based renal and more recently hepatic denervation were proven to be safe and demonstrated significant improvements in the cardiometabolic profile in line with the pathophysiological considerations described earlier such as the hepato-renal reflex and the close interaction between the SNS and glucose metabolism [125,126]. Increased sympathetic tone in the vasculature of the skeletal muscle plays a significant role in glucose metabolism, mainly through reduction in blood flow to the skeletal musculature, thereby diminishing glucose uptake, a hallmark of insulin resistance [9]. Renal sympathetic denervation substantially reduced sympathetic activity [127,128,129] and improved glucose metabolism along with BP lowering in patients with resistant hypertension [130]. Renal denervation performed in woman with polycystic ovary syndrome, commonly characterized by overweight/obesity, insulin resistance, sympathetic overdrive and BP elevation, improved insulin sensitivity by 17.5% in the absence of any weight changes at three months accompanied by regression of renal damage [131,132].

Additionally, attenuation of sympathetic tone in the liver, pancreas and duodenum through hepatic denervation modulates the cardiac and renal sympathetic activity and may offer far-reaching cardiovascular and metabolic benefits [133,134,135,136]. Hepatic denervation attenuates the central sympathetic outflow through afferent pathways from liver to brain, which may influence other autonomic nervous control and therefore contribute to improvements in blood pressure and metabolism [125]. The COMPLEMENT trial (NCT02278068) trial, a first in human (FIH) feasibility study, conducted in New Zealand, demonstrated that hepatic sympathetic denervation was safe and effective, resulting in >0.5% HbA1c reduction in uncontrolled diabetes mellitus (*n* = 46) along with blood pressure lowering (unpublished data). Other interventional sympatholytic techniques include the baroreflex activation therapy that showed modest metabolic benefits in patients with resistant hypertension [137,138].

Despite multiple available approaches to target the various aspects of the cardio metabolic disease spectrum, the prevalence of MAFLD continues to remain high, the control rates remain unacceptably low and alternative definitive therapeutic approaches are warranted. Whether the interventional sympatholytic strategies can provide a solution for MAFLD is yet to be tested in future clinical trials. However, across the metabolic disease continuum, where the central pathogenesis is identified to be “sympathetic overdrive” in disease states such as MAFLD, obesity, MetS and hypertension, it seems relevant to target the underlying pathology rather than the cluster of abnormalities resulting from heightened sympathetic activation individually.

## 9. Conclusions

**Figure 4:**
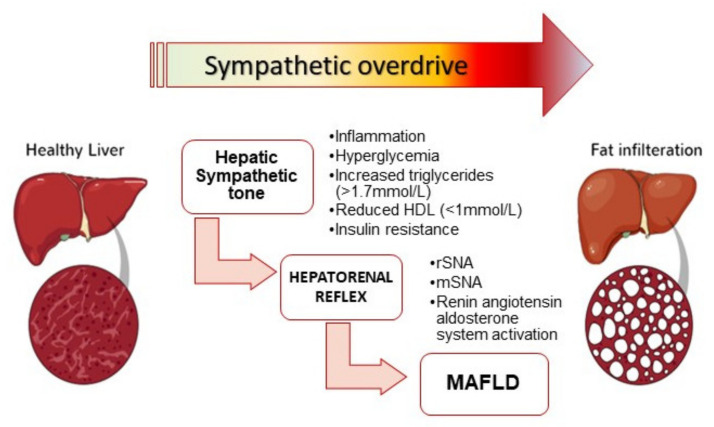
MAFLD, a consequence of enhanced hepatic sympathetic tone: Sympathetic activation reduces hepatic artery flow, induces hepatic hypoxia, impairs hepatic artery compliance and increases hepatic resistance from hepatocyte. The resulting decrease in the hepatic ATP leads to the accumulation of adenine nucleotides and stimulates the hepato-renal reflex sustaining feed forward sympathetic activation between the liver and kidney. MAFLD is both a cause and effect of the increased hepatic sympathetic tone, a hepatic manifestation of metabolic syndrome.

Accumulating evidence from animal and clinical studies substantiates an important role of the sympathetic nervous system in the perturbation of the cardiovascular and metabolic homeostasis. With use of state-of-the-art methods, enhanced sympathetic activation has been clearly demonstrated both in animals and in humans with obesity, MetS and MAFLD. Furthermore, the cardiovascular and metabolic derangements commonly associated with the metabolic disease spectrum, such as elevated blood pressure, diastolic dysfunction and renal impairment, are also modulated by the sympathetic nervous system. Mechanistically, sympathetic activation affects relevant aspects of the metabolic perturbations that underlie MAFLD and metabolic effects, occurring in response to increased hepatic sympathetic tone (Figure 4). Although the exact nature of the association between sympathetic activation and MAFLD remains to be determined, there is evidence to suggest an important role of the sympathetic nervous system in the onset and progression of MAFLD. Common management strategies for MetS such as weight loss and exercise have been associated with significant reduction in sympathetic activation. Targeting the sympathetic nervous system directly—either with pharmacotherapy or with novel device-based strategies—seems to be a rational and attractive next step, particularly with the escalating prevalence of obesity and MetS since many of these patients find dietary and exercise regimens unsustainable. Further studies are needed to validate the role of the sympathetic nervous system as a useful therapeutic target in MAFLD and other metabolic diseases.

## Figures and Tables

**Table 1:** Dietary and pharmacotherapy considerations in MAFLD.

Intervention Strategy	Mechanism of Action	Outcomes
**Pharmacotherapeutic Strategies**		
Vitamin E (*α*-tocopherol)	Free radical scavenger—inhibits oxidative stress	Reductions in serum aminotransferases levels and improvement in hepatic inflammation resolution of steatohepatitis [89,90]. However, vitamin E supplement (400 IU/day) was associated with all cause mortality [91] and prostate cancer [92]
Metformin	Insulin sensitizer	Varied outcomes with improvement in hepatocellular inflammation, steatosis and fibrosis, however inconclusive [2,93,94,95]
Thiazolidinediones (TZDs), e.g., pioglitazone	Enhanced insulin sensitivity by acting on peroxisome proliferator-activated receptor gamma and increasing circulating adiponectin prevent the activation of adipocyte c-jun kinase, a kinase that when activated impairs adipocyte responsiveness to insulin and adipocyte storage of TG [96,97,98]	Significantly improved aminotransferase levels, hepatic inflammation and steatosis but did not alter the stage of fibrosis [57,65,96,97] The long-term use of TZDs is associated with side effects such as weight gain (average 4 Kg),congestive heart failure (CHF), other cardiovascular morbidity, bone loss (fracture risk) and urinary bladder cancers [99]
Statins	Inhibitors of cholesterol synthesis	Lack of evidence and increased risk of drug induced liver injury [75]
Weight loss medication	Weight loss mediated beneficial effect on MAFLD	No medication for weight loss has yet been identified, to have long-term safety, efficacy and tolerability [100]
**Dietary interventions**		
*n* − 3/*n* − 6 polyunsaturated fatty acids (PUFA) dietary ratio	low *n* − 3/*n* − 6 PUFA ratio in MAFLD	Supplementation of omega-3 [101] and other PUFAs in diet [102] have shown a beneficial effect on both hepatic lipogenesis and steatosis [101,102,103]
Trans-fat enriched foods High fructose foods, e.g., corn syrup		Insulin resistance Hepatic steatosis hepatic fructose metabolism favors ATP depletion, lipotoxicity, insulin resistance and enhances enhanced TNF expression [104]
Coffee (caffeine)	Caffeine alters TGF*β* signaling pathways to reduce the transcription of connective tissue growth factor (CTGF), a major stimulator of fibrosis [105,106,107].	Reduction of hepatic inflammation and fibrosis in morbidly obese MAFLD patients [105,106,107]

## Data Availability

Not applicable.
